# Treatment response to indacaterol/glycopyrronium versus salmeterol/fluticasone in exacerbating COPD patients by gender: a post-hoc analysis in the FLAME study

**DOI:** 10.1186/s12931-019-0972-7

**Published:** 2019-01-08

**Authors:** Jadwiga A. Wedzicha, Dave Singh, Ioanna Tsiligianni, Christine Jenkins, Sebastian Fucile, Robert Fogel, Steven Shen, Pankaj Goyal, Karen Mezzi, Konstantinos Kostikas

**Affiliations:** 1 Respiratory Division, National Heart and Lung Institute, Imperial College London, London, UK; 2 Division of Infection, Immunity and Respiratory Medicine, University of Manchester, Manchester, UK; 3 Department of Social Medicine, Faculty of Medicine, University of Crete, Heraklion, Crete Greece; 4 The George Institute for Global Health, Sydney, Australia; 5 Novartis Pharmaceuticals Corporation, East Hanover, NJ USA; 6 Novartis Pharma AG, Basel, Switzerland; 7 Respiratory Medicine Department, University of Ioannina Medical School, Ioannina, Greece

**Keywords:** Chronic obstructive pulmonary disease, Gender, Indacaterol/glycopyrronium, Exacerbation reduction, Lung function

## Abstract

**Background:**

The burden of chronic obstructive lung disease (COPD) is increasing in women, with recent evidence suggesting gender differences in disease characteristics and potentially in treatment outcomes.

**Methods:**

FLAME was a 52-week randomized controlled trial in patients with severe-to-very-severe COPD and a history of exacerbations. In this post-hoc analysis, gender-based baseline differences and treatment outcomes between indacaterol/glycopyrronium 110/50 μg once daily (IND/GLY) and salmeterol/fluticasone 50/500 twice daily (SFC) were assessed in terms of rate of exacerbations, time-to-first exacerbation, lung function, health status, and rescue medication use.

**Results:**

This post-hoc analysis included 2557 men and 805 women. Baseline characteristics differed between genders, with women being younger, having better lung function and more often experiencing ≥2 exacerbations in the previous year. Compared with SFC, IND/GLY treatment was associated with reductions in the annualized rates of moderate/severe exacerbations (rate ratio [95% CI]: 0.81 [0.73–0.91], 0.89 [0.74–1.07] in men and women, respectively). Similarly, time-to-first moderate/severe exacerbation was also delayed (hazard ratio [95% CI]: 0.79 [0.70–0.89] and 0.76 [0.63–0.91] in men and women, respectively). Results were similar for all (mild/moderate/severe) exacerbations. Improvements in lung function, health status and rescue medication use with IND/GLY vs SFC were comparable between men and women. The smaller sample size for women may account for some observed discrepancies in treatment responses.

**Conclusions:**

Although there were gender differences in baseline characteristics, IND/GLY demonstrated similar trends for exacerbation prevention and lung function improvement in men and women with moderate-to-very-severe COPD and a history of exacerbations compared with SFC. Small differences in the effects seen between genders may be attributed to the different sizes of the two groups and need to be further evaluated in randomized trials that are appropriately powered for gender analysis.

**Trial registration:**

Post hoc analysis of the FLAME study. ClinicalTrials.gov number: NCT01782326. Registered 1 February 2013.

## Background

While long considered primarily a disease of older men, recent data have shown an increase in the prevalence and burden of chronic obstructive pulmonary disease (COPD), along with a considerable rise in mortality, in women [1, 2]. In fact, in some countries, COPD-related mortality in women has surpassed that of men [1]. Additionally, evidence from available studies suggests that women have greater airway hyper-responsiveness, increased dyspnea for a given level of airflow limitation, greater frequency of exacerbations and worse health status compared with men [3–8]. These differences may be due to biological, anatomical or psychological factors, and might influence disease management and treatment response [1, 9–11].

Historically, many more men than women participate in randomized clinical trials in COPD, and the increasing prevalence of COPD in women is only slowly being reflected in trial populations, in contrast to real life [12]. Hence, limited data are available on the influence of gender on treatment effectiveness in COPD. A recent pooled analysis of six trials from the IGNITE program reported that treatment with indacaterol/glycopyrronium (IND/GLY) was similarly efficacious in women and men with moderate-to-very-severe COPD in terms of improvement in lung function and health status, and reduction in dyspnea and rescue medication use [7]. Although robust, this analysis involved relatively few patients with very severe COPD or a history of exacerbations at baseline. Thus, there remains a gap in our knowledge of whether gender characteristics impact overall treatment response, especially in terms of exacerbation rate reduction.

The FLAME study demonstrated superiority of IND/GLY over salmeterol/fluticasone (SFC) in preventing COPD exacerbations in moderate-to-very-severe COPD patients with a prior history of exacerbations [13]. In this post-hoc analysis, we evaluated gender-based differences in the FLAME study population in terms of baseline characteristics and efficacy outcomes.

## Methods

FLAME was a 52-week, randomized, double-blind, double-dummy, active-comparator study in patients with moderate-to-very-severe COPD who received either IND/GLY 110/50 μg once daily (o.d.) via Breezhaler® or SFC 50/500 μg twice daily (b.i.d.) via Accuhaler® (ClinicalTrials.gov number: NCT01782326) [13]. This post-hoc analysis assessed gender-based differences in baseline characteristics and efficacy outcomes in patients treated with IND/GLY vs SFC in terms of reduction in annual rate of moderate/severe and all (mild/moderate/severe) COPD exacerbations, changes in lung function, health status and reduction in rescue medication use, during 52 weeks of treatment.

### Patients

Key inclusion criteria for the FLAME study were COPD patients aged ≥40 years with post-bronchodilator forced expiratory volume in 1 s (FEV_1_) from 25 to < 60% of predicted normal, FEV_1_/forced vital capacity (FVC) < 0.70 and a history of ≥1 exacerbation in the past year [13]. Key exclusion criteria were patients with COPD exacerbations requiring treatment with antibiotics, systemic corticosteroids and/or hospitalization 6 weeks prior to screening, any history of asthma, blood eosinophil counts > 600/mm^3^ at the end of screening and a history of long QT syndrome or prolonged corrected QT (> 450 ms) at the start of run-in [13]. Detailed inclusion and exclusion criteria are available in the primary publication [13]. The full analysis set from the FLAME study was used for the analysis of outcomes.

### Assessments

Differences between men and women in baseline demographic and clinical characteristics including age, inhaled corticosteroid (ICS) use, COPD severity, and pre- and post-bronchodilator FEV_1_ were analyzed. All efficacy outcomes assessed in this post-hoc analysis were consistent with those analyzed in the primary FLAME study [13]. Comparison of efficacy outcomes between treatment with IND/GLY and SFC at Week 52 in men and women included assessment of annualized rates of moderate/severe and all COPD exacerbations, time-to-first moderate/severe and all exacerbation, trough FEV_1_, health status (St. George’s Respiratory Questionnaire for COPD [SGRQ-C] total score), and rescue medication use. Data for rescue medication use were obtained from an electronic diary (e-Diary). COPD symptoms were captured using the e-Diary, which also sent an alert to the patients and physicians to the presence of potential exacerbations. During visits to the clinic, exacerbation data were based on the electronic case report form and assessment by a healthcare professional. Exacerbations, which were defined symptomatically according to the criteria of Anthonisen et al. [14] and based on healthcare resource utilization, were categorized as mild (involving worsening of symptoms for > 2 consecutive days but not leading to treatment with systemic glucocorticoids or antibiotics), moderate (leading to treatment with systemic glucocorticoids, antibiotics, or both), or severe (leading to hospital admission or a visit to the emergency department that lasted > 24 h in addition to treatment with systemic glucocorticoids, antibiotics, or both). The primary outcome of the annualized rate of all exacerbations in the FLAME study refers to the sum of mild, moderate and severe exacerbations. The proportion of patients achieving the minimal clinically important difference (MCID; ≥4 unit reduction) in SGRQ-C total score was also assessed.

### Statistical analysis

This was a post-hoc analysis, not pre-specified in the statistical analysis plan of the FLAME study that included baseline data from the 3362 randomized patients and treatment outcomes data, depending on availability, from the full analysis set of 3354 patients from the FLAME study. Descriptive statistics were used to summarize gender-wise demographic and clinical characteristics. Comparison between genders used two-sample *t*-tests for continuous variables and chi-square tests (or Fisher’s exact test as appropriate) for categorical variables. Differences in the treatment outcome measures in men and women from baseline to Week 52 were assessed using a mixed model for repeated measures or a logistic regression model with treatment, baseline value of measure, smoking status at screening, ICS use at screening, airflow limitation severity, visit, and gender interaction as fixed effects. Treatment differences between IND/GLY and SFC for each gender are expressed as least squares mean (LSM) from the above-mentioned model, 95% confidence interval (CI) and *P* values. Kaplan-Meier plot and Cox regression models with the same model terms as above were used to estimate the time-to-first exacerbation. All statistical analyses were performed using Statistical Analysis Software (SAS) with *P* values < 0.05 considered statistically significant.

## Results

### Demographic characteristics

Of the 3362 patients randomized in the FLAME study, 2557 (76%) were men. At baseline, women were significantly younger, had higher body mass index (BMI) and longer duration of COPD compared with men. Although a higher proportion of women than men were current smokers, women were found to have a lower cumulative smoking habit. A greater number of men had more severe COPD and airflow limitation than women at baseline; however, a higher proportion of women had a history of ≥2 exacerbations in the previous year. The degree of dyspnea (modified Medical Research Council [mMRC] grade) at baseline was similar between men and women, but COPD assessment test (CAT) score was higher in women. In terms of lung function at baseline, women had better lung function, as expressed by higher values of % predicted post-bronchodilator FEV_1_ and FEV_1_/FVC ratio, and similar post-bronchodilator FEV_1_ reversibility. At baseline, greater proportions of women were using ICS (63.5% vs 54.0%) and long-acting β_2−_agonist (LABA; 75.3% vs 64.6%) compared with men (Table 1).

**Table 1:** Demographic and clinical characteristics at baseline

	Men *n* = 2557	Women *n* = 805	*P* value
Age, years	65.2 ± 7.7	62.6 ± 7.6	< 0.001
Duration of COPD, years	7.1 ± 5.2	7.7 ± 5.9	0.004
Current smoker, *n* (%)	946 (37.0)	387 (48.1)	< 0.001
Estimated number of pack years	43.6 ± 22.5	36.0 ± 18.5	< 0.001
Severity of COPD (GOLD 2015), *n* (%)			
Low risk and more symptoms (Group B)	589 (23.0)	233 (28.9)	0.002
High risk and more symptoms (Group D)	1945 (76.1)	569 (70.7)	
Severity of airflow limitation (GOLD 2011–2014), *n* (%)			
Moderate (GOLD 2)	804 (31.4)	319 (39.6)	< 0.001
Severe (GOLD 3)	1506 (58.9)	448 (55.7)	
Very severe (GOLD 4)	222 (8.7)	35 (4.3)	
Pre-bronchodilator FEV_1_, L	1.1 ± 0.3	0.8 ± 0.2	< 0.001
Post-bronchodilator FEV_1_, L	1.3 ± 0.3	1.0 ± 0.3	< 0.001
Post-bronchodilator FEV_1_, % predicted	43.5 ± 9.5	45.8 ± 9.0	< 0.001
Post-bronchodilator FEV_1_ reversibility, % of baseline value	22.2 ± 16.0	22.9 ± 16.2	0.233
Post-bronchodilator FEV_1_/FVC, %	40.9 ± 9.9	43.7 ± 9.5	< 0.001
Number of COPD exacerbations in the previous year, n (%)			
1	2094 (81.9)	616 (76.5)	0.002
≥ 2	461 (18.0)	188 (23.4)	
CAT score^a^	16.5 ± 7.0	17.7 ± 7.1	< 0.001
mMRC^b^, *n* (%)			
Grade 0–1	4 (0.2)	1 (0.1)	0.954
Grade 2	1838 (71.9)	574 (71.3)	
Grade 3	660 (25.8)	211 (26.2)	
Grade 4	55 (2.2)	19 (2.4)	
BMI (kg/m^2^)	25.7 ± 4.9	26.3 ± 6.0	0.003
ICS use, *n* (%)	1382 (54.0)	511 (63.5)	< 0.001
LAMA use, *n* (%)	1535 (60.0)	502 (62.4)	0.238
LABA use, *n* (%)	1651 (64.6)	606 (75.3)	< 0.001

Demographic and clinical characteristics at baseline for the randomized set has been presented. Data are presented as mean ± SD unless otherwise stated. *P-*values are based on *t*-tests for continuous variables and chi-square tests (or Fisher’s exact test as appropriate) for categorical variables. ^a^on a scale of 0–40, with higher scores indicating worse health status; ^b^on a scale of 0–4, with higher scores indicating more severe dyspnea

*BMI* body mass index, *CAT* COPD assessment test, *COPD* chronic obstructive pulmonary disease, *FEV*_*1*_ forced expiratory volume in 1 s, *FVC* forced vital capacity, *GOLD* Global Initiative for Chronic Obstructive Lung Disease, *ICS* inhaled corticosteroid, *LABA* long-acting β_2_-agonist, *LAMA* long-acting muscarinic antagonist, *mMRC* modified Medical Research Council, *SD* standard deviation

### Efficacy of treatment by gender

**Fig. 1:**
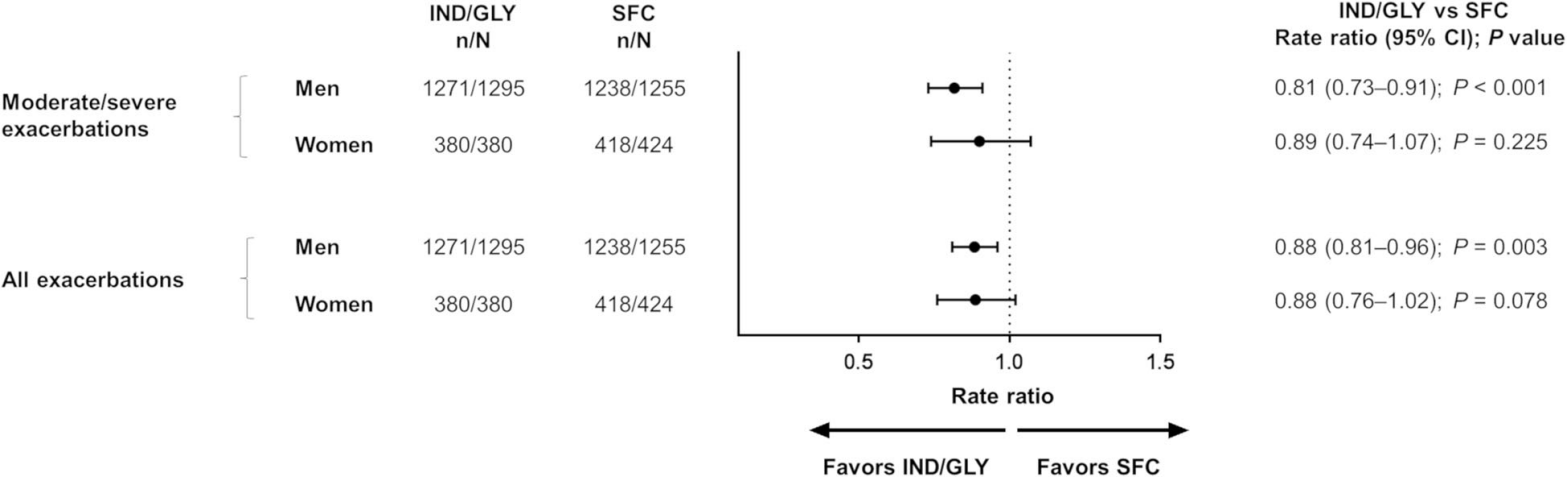
Annualized rate of moderate/severe and all (mild/moderate/severe) exacerbations. n, number of patients included in the analysis; N, total number of patients in the study. b.i.d., twice daily; CI, confidence interval; IND/GLY, indacaterol/glycopyrronium 110/50 μg o.d.; o.d., once daily; SFC, salmeterol/fluticasone 50/500 μg b.i.d

The annualized rates of moderate/severe exacerbations were lower in both men and women treated with IND/GLY (rate ratio [RR, 95% CI]: 0.81 [0.73–0.91] and 0.89 [0.74–1.07], respectively) compared with SFC. Similarly, the rates of all exacerbations were lower in both IND/GLY-treated men and women (RR [95% CI]: 0.88 [0.81–0.96] and 0.88 [0.76–1.02], respectively) compared with SFC (Fig. 1).

**Fig. 2:**
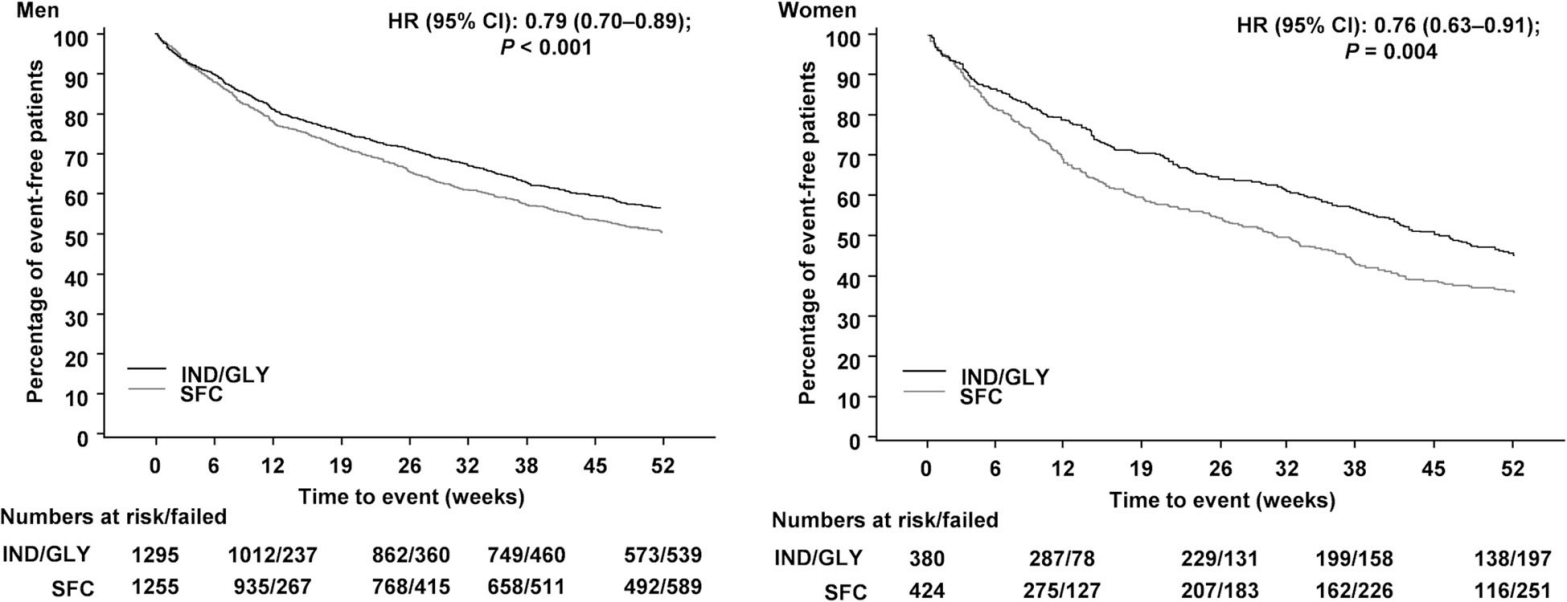
Time-to-first moderate/severe COPD exacerbations. The analyses were performed in the full analysis set from the FLAME study. Subjects who did not experience a COPD exacerbation were censored at the earlier date of last double-blind treatment + 1, death and final visit. Figure truncated after Week 52 (Day 365). b.i.d., twice daily; CI, confidence interval; HR, hazard ratio; IND/GLY, indacaterol/glycopyrronium 110/50 μg o.d.; o.d., once daily; SFC, salmeterol/fluticasone 50/500 μg b.i.d

**Fig. 3:**
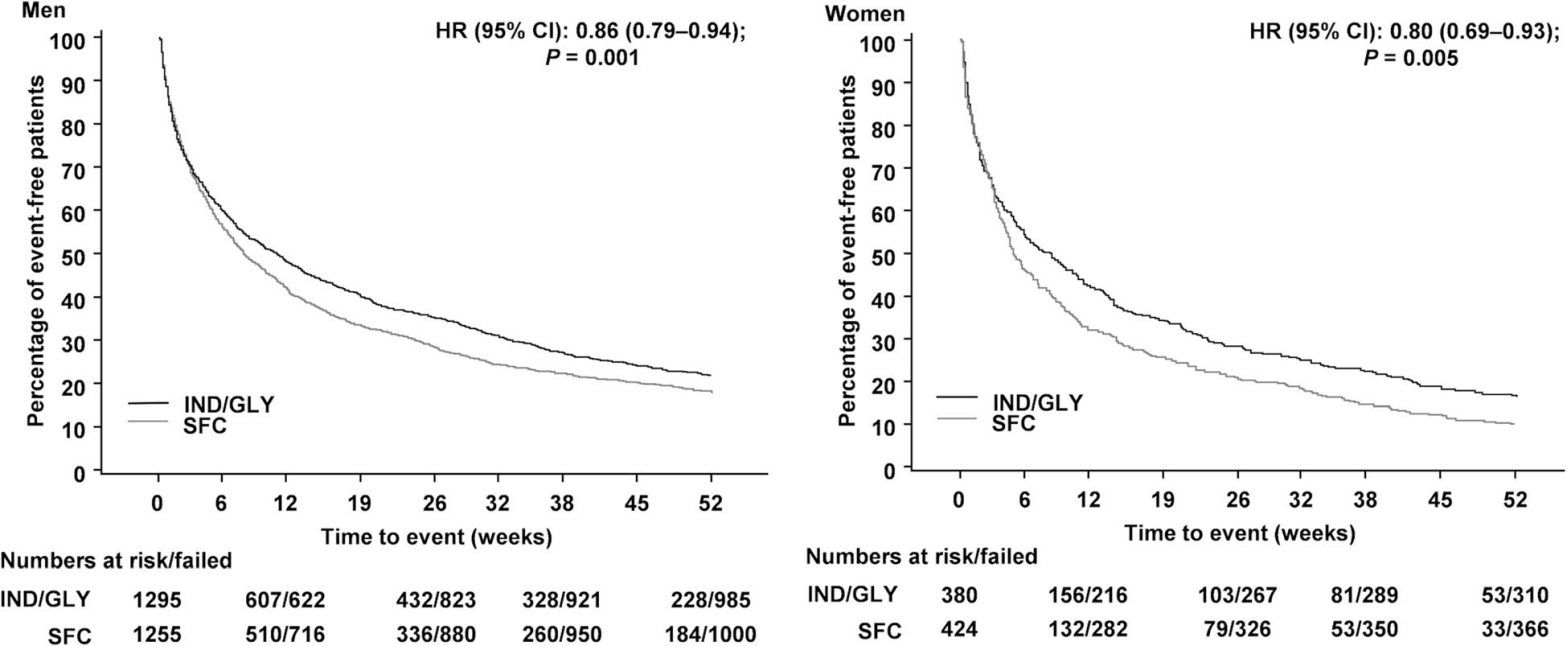
Time-to-first all (mild/moderate/severe) COPD exacerbations. The analyses were performed in the full analysis set from the FLAME study. Subjects who did not experience a COPD exacerbation were censored at the earlier date of last double-blind treatment + 1, death and final visit. Figure truncated after Week 52 (Day 365). b.i.d., twice daily; CI, confidence interval; HR, hazard ratio; IND/GLY, indacaterol/glycopyrronium 110/50 μg o.d.; o.d., once daily; SFC, salmeterol/fluticasone 50/500 μg b.i.d

Time-to-first moderate/severe exacerbation was delayed in IND/GLY-treated men (hazard ratio [HR, 95% CI]: 0.79 [0.70–0.89]) and women (HR [95% CI]: 0.76 [0.63–0.91]) compared with men and women receiving SFC (Fig. 2). Similarly, time-to-first exacerbation for all (mild, moderate and severe) exacerbations was delayed in IND/GLY-treated men (HR [95% CI]: 0.86 [0.79–0.94]) and women (HR [95% CI]: 0.80 [0.69–0.93]) compared with those receiving SFC (Fig. 3).

In both men and women, the change from baseline in pre-dose FEV_1_ was higher in IND/GLY-treated patients compared with those receiving SFC (LSM men: 67 mL; women: 42 mL). Improvement in health status (SGRQ-C total score) was higher in men and numerically higher in women treated with IND/GLY (LSM, − 1.3 and − 1.1, respectively) compared with those receiving SFC (Table 2).

**Table 2:** Change from baseline: IND/GLY vs SFC in men and women with COPD

	Treatment difference (IND/GLY vs SFC)			
	Men *n* = 2550	*P* value	Women *n* = 804	*P* value
Trough FEV_1_ (mL) (LSM [95% CI])	67 (51–84)	< 0.001	42 (12–71)	0.006
SGRQ-C total score (LSM [95% CI])	−1.3 (−2.3– −0.4)	0.007	−1.1 (−2.8–0.7)	0.230
Proportion of patients with MCID (≥4 units) in SGRQ-C (OR [95% CI])	1.29 (1.09–1.54)	0.004	1.32 (0.96–1.83)	0.093
Rescue medication use (puffs/day) (LSM [95% CI])	−0.27 (− 0.43– − 0.12)	< 0.001	− 0.18 (− 0.45–0.09)	0.189

The analyses were performed in the full analysis set from the FLAME study, based on data availability. Data are presented as LSM. *b.i.d.* twice daily, *CI* confidence interval, *COPD* chronic obstructive pulmonary disease, *FEV*_*1*_ forced expiratory volume in 1 s, *IND/GLY* indacaterol/glycopyrronium 110/50 μg o.d., *LSM* least squares mean, *MCID* minimal clinically important difference, *o.d.* once daily, *OR* odds ratio, *SFC* salmeterol/fluticasone 50/500 μg b.i.d., *SGRQ-C* St. George’s Respiratory Questionnaire for COPD

Higher proportions of men (49.8% vs 43.5%; odds ratio [OR, 95% CI]: 1.29 [1.09–1.54]) and women (47.5% vs 44.6%; OR [95% CI]: 1.32 [0.96–1.83]) treated with IND/GLY compared with those treated with SFC achieved ≥4-unit improvement in SGRQ-C total score. The reduction in rescue medication use from baseline was higher in IND/GLY-treated men and women (LSM men: − 0.27, women: − 0.18) compared with those receiving SFC (Table 2).

## Discussion

One key finding of this gender-based post-hoc analysis of the FLAME study was that at baseline, women were younger, more often current smokers, had poorer health status, experienced exacerbations more frequently despite having similar or slightly better lung function, and were treated more often with ICS than men. Nevertheless, despite differences in baseline characteristics in women and men, 52 weeks of treatment with IND/GLY presented similar trends for exacerbation prevention, and improvement in in lung function, health status and use of rescue medication compared with SFC, in both men and women. These findings further support the efficacy of IND/GLY in patients with moderate-to-very-severe COPD with a history of exacerbations, regardless of gender. This is the first analysis to our knowledge on the efficacy of a LABA/LAMA vs a LABA/ICS in moderate-to-very-severe exacerbating COPD patients.

Limited data are available on the treatment efficacy of dual bronchodilators by gender especially in comparison with ICS/LABA therapies; data from a previous analysis of the IGNITE program indicate comparable efficacy of IND/GLY in both genders compared with placebo and active comparators [7]. Moreover, the studies that assessed the differential response of bronchodilators reported inconsistent findings. A report on the LAMA tiotropium suggested that men and women had comparable treatment efficacy in terms of lung function and health status [15]. The Lung Health Study showed a differential response of ipratropium, a short-acting muscarinic antagonist, in women and men with mild-to-moderate COPD with larger effect size in women. These findings suggest that muscarinic antagonists may show a differential therapeutic response, possibly as a result of altered expression of muscarinic M3 and M2 receptors between women and men [11, 16]. In the TORCH study [5], although women had lower mortality than men, they had greater incidence of exacerbations with worsening dyspnea and health status. Another analysis of the TORCH study reported that treatment with SFC slowed the rate of decline in lung function equally in both genders, as compared to its monocomponents, or placebo [17]. In the TRISTAN study [18], similar improvement in lung function and health status, and reduction in exacerbations was seen in men and women treated with SFC. The findings from the present analysis however reinforce the results of IGNITE post-hoc analysis [7] and support IND/GLY as a more effective treatment option in terms of improvement in lung function and reduction in exacerbation compared with SFC in both men and women.

At baseline, women with COPD differed significantly from men in their clinical characteristics, with more women being current smokers. Data from the FLAME study also supports other recent evidence that women with COPD are generally younger than men with a greater proportion being current smokers despite the lower number of pack-years reported [1]. The analysis of data from six clinical trials from the IGNITE program [7] and real-world studies such as DACCORD [8] reported similar findings [2]. This is consistent with other studies [2, 7, 8] with data suggesting that women are less likely to succeed with smoking cessation [19]. Previous studies comparing current vs ex-smokers treated with IND/GLY and SFC have shown some differential efficacy in terms of lung function between treatments [20, 21]. In the FLAME study population, women had less severe COPD as measured by FEV_1_, and a higher proportion had a history of ≥2 exacerbations, and worse health status (CAT score) at baseline than men. Similar differences in baseline characteristics between genders, with women being more symptomatic and having a greater history of exacerbations, were reported in the IGNITE program [7], ARCTIC [9], DACCORD [8] and TORCH [5] studies. Other studies have suggested that women tend to present with exacerbations more often than men, which could explain the less severe COPD but higher incidence of exacerbations [22, 23].

Assessment of health status is a key component in the management of COPD as it reflects the impact of the disease on quality of life. Over the past decade, evaluation of symptom burden using CAT in patients with COPD has gained precedence in clinical settings due to its ease of use and its relationship with severity of exacerbation [24–27]. The higher CAT scores at baseline in women than in men observed in our analysis could reflect the greater impact of symptoms in women. In addition, psychological factors, such as anxiety and depression, are associated with increased exacerbation risk in COPD [28] and such conditions are more prevalent in women with COPD [29–31]. This may partly account for the higher exacerbation rates reported by women at baseline.

Studies suggest that women presenting with COPD symptoms are often misdiagnosed with asthma, potentially due to lack of spirometry testing at diagnosis [2, 32, 33], which may account for the high ICS use observed here at baseline. In addition, the higher symptom burden and exacerbation frequency in women at baseline may also account for increased ICS prescription rates. This potential overuse of ICS may be associated with numerous consequences including an increased risk of fractures [34], highlighting the need for alternative treatment/reduction of ICS use in older women with COPD. Women could be more vulnerable to the adverse effects of ICS, as for example, women are more prone to osteoporosis at a younger age than men. However, differences in adverse effects have not been demonstrated to date. Moreover, severe airflow obstruction in COPD patients is an important risk factor for osteoporosis [35, 36], a disease that is more prevalent in women than men. [37, 38] Baseline data from this analysis together with the ARCTIC [9], DACCORD [8] and TORCH [5] studies indicate that women experience exacerbations more frequently than men, hence making prevention of exacerbation an important treatment objective in women. The current findings emphasize the differences in clinical presentation of COPD between genders and support the need for improved understanding of the disparity in pathophysiology and presentation of COPD between genders. With the increasing prevalence of COPD in women, an improved understanding, especially in real-life settings, is important to aid diagnosis and tailoring disease management strategy that is specific to women [1, 2, 12].

During the 4-week run-in period in the FLAME study when all patients received tiotropium alone, only 3.6% of patients discontinued treatment because of exacerbations or lung function deterioration at randomization [13]. This fact minimizes the potential bias of the standardized treatment with tiotropium during run-in. In this analysis, both men and women responded better to IND/GLY compared with SFC. The majority of the patients in the FLAME study had mMRC score ≥ 2 at baseline and were thus categorized as GOLD group B and D, having higher rate of exacerbations with more symptoms and poorer health status.

It is important to note that effect size in women and men was not always similar in this analysis, which may be due to the smaller sample size of women in the study. Moreover, this is a post-hoc analysis and all results should be interpreted with caution compared to the pre-specified analyses in the original study [39]. The fact that the two groups are clearly defined and all patients from the primary study population have been included minimizes the risk for potential biases. Yet, given the fact that the FLAME study was not powered for these subgroup analyses, all the results reported here should be considered as hypothesis-generating and there is a need for further studies specifically designed to assess gender-related differences in treatment outcomes. Nevertheless, the magnitude of the treatment differences was generally quite consistent between genders, suggesting that IND/GLY is efficacious in both men and women with COPD.

## Conclusions

This post-hoc gender-based analysis of the FLAME study demonstrated that despite some significant differences in baseline characteristics between the genders, IND/GLY demonstrated similar trends for exacerbation prevention and improvement in lung function, health status and use of rescue medication in men and women with moderate-to-very-severe COPD and a history of exacerbations compared with SFC. Small differences in the effects seen between genders may be due to the different sample sizes of the two groups and need to be evaluated in randomized trials that are appropriately powered for gender analysis.

## Abbreviations

b.i.d.: twice daily
BMI: Body mass index
CAT: COPD assessment test
CI: Confidence interval
COPD: Chronic obstructive lung disease
e-Diary: Electronic diary
FEV_1_: Forced expiratory volume in 1 s
FVC: Forced vital capacity
HR: Hazard ratio
ICS: Inhaled corticosteroid
IND/GLY: Indacaterol/glycopyrronium 110/50 μg o.d
LABA: Long-acting β_2_-agonist
LAMA: Long-acting muscarinic antagonist
LSM: Least squares mean
MCID: Minimal clinically important difference
mMRC: modified Medical Research Council
o.d.: once daily
OR: Odds ratio
RR: Rate ratio
SFC: Salmeterol/fluticasone 50/500 μg b.i.d
SGRQ-C: St. George’s Respiratory Questionnaire for COPD
